# Feasibility and acceptability of a mental health stigma intervention for low-income South African caregivers: A qualitative investigation

**DOI:** 10.4102/sajpsychiatry.v28i0.1824

**Published:** 2022-03-29

**Authors:** Portia Monnapula-Mazabane, Inge Petersen

**Affiliations:** 1 Discipline of Psychology, School of Applied Human Sciences, University of KwaZulu-Natal, Durban, South Africa; 2 Centre for Rural Health, School of Nursing and Public Health, University of KwaZulu-Natal, Durban, South Africa

**Keywords:** acceptability, feasibility, intervention, mental health stigma, caregivers, burden of care, coping

## Abstract

**Background:**

Common mental health illnesses such as depression and anxiety disorders are increasing globally. There remain significant gaps in health services provision and support for mental illness linked to stigma in developing countries.

**Aim:**

Our study aimed to assess the feasibility and acceptability of a mental health intervention for caregivers of mental health service users.

**Setting:**

Low-income South African communities.

**Method:**

Our study qualitatively assessed the feasibility of an anti-stigma mental health intervention for family caregivers in low-income settings. The intervention was structured into five sessions delivered over three days. Caregivers attended all the sessions at a centralised community venue. Semi-structured qualitative interviews were held separately with caregivers (*n* = 10) and their service users (*n* = 9) eight weeks post-intervention. Interviews were translated verbatim from local languages to English prior to framework analysis.

**Results:**

Post-intervention, service users reported improved family relations and understanding of mental illness among family members. The intervention was reported as acceptable and helpful by caregivers as it increased knowledge, fostering better relationships with service users. Group discussions were noted as a critical driver of intervention success. Widespread mental health stigma within communities remained a key concern for caregivers and service users.

**Conclusion:**

With the government’s drive for deinstitutionalisation, the need to integrate anti-stigma interventions within community mental health services is vital, as is the need for population-wide anti-stigma interventions to support the integration of mental health service users within communities.

## Introduction

The mental health burden is increasing in Africa and is estimated to be responsible for 9% of the non-communicable diseases burden.^1^ Common mental health illnesses such as depression and anxiety disorders contribute about 8% and 3%, respectively, to the years lost to disability in Africa.^2^ Currently, mental illnesses are responsible for 13.6 million disability-adjusted life years (DALYs) in the region.^1^ Despite this surge in mental health burden, there is an estimated mental health treatment gap of 76% – 85% for serious mental disorders in low- and middle-income countries (LMICs).^3^ In South Africa, an estimated gap of 75% for common mental disorders (anxiety, depressive and substance use disorders) has been reported.^4^

Multiple factors are attributed to Africa’s large mental illness treatment gap, including low priority at a policy level, lack of skilled personnel, limited financing, and poor allocation of financial resources towards institutional care.^5,6^ Institutional mental health stigma plays a role in the low priority of mental health in resource allocation, contributing to limited progress towards universal access to mental health services globally.^7^ In Africa, limited service availability is compounded by low service demand fuelled by traditional beliefs and misconceptions that promote mental health stigma. Consequently, this results in a cycle of poor mental health services utilisation, demand availability and funding for services.^7,8,9^

Due to the limited mental health services in Africa,^5^ caregiving for service users with severe mental illness often falls onto family members who are the only available support.^10,11,12^ Caregiving is an immense and demanding burden, and families are often left to cope on their own. The South African health system offers no specialised/integrated support for family caregivers while there are no limits to the specialist services [at tertiary level facilities] available to service users. Furthermore, stigma against the family of an individual with mental illness is common in Africa,^13^ and it provides an added burden of negotiating social stigmatisation. Due to mental health stigma, both mental health service users and their family caregivers suffer from low self-esteem, shame, anger and this often results in attempts to conceal the stigma.^14^

Stigma limits the uptake of mental health services and integration of people with mental illness into communities and society at large.^7^ In South Africa, caregivers and service users report stigma experiences within families and communities, resulting in families withholding the information that a family member has a mental illness from community members for fear of stigmatisation.^15^ High levels of mental health stigma within communities threaten the social reintegration of service users and the deinstitutionalisation of mental health services and care – which are central to the South African National Mental Health Policy Framework (2013–2020). Ironically, a shift from institutional to community mental health care approaches are meant to counter the psychiatric stigma associated with institutionalisation that removed people with mental health illness from society.^16^

Studies in high-income countries have demonstrated short- to medium-term improvements in mental health knowledge and a few reported attitudinal improvements.^17^ Variances in mental health stigma intervention findings are attributed to the differences in study design, delivery approaches and target populations.^17,18^ Research on the interventions to reduce mental health stigma within communities remains sparse in developing countries. Over half of the published studies on stigma reduction interventions in developing countries are focused on human immunodeficiency virus (HIV) and only three studies concentrate on mental health.^19^ Only one study was identified that focused on addressing community mental health stigma in African settings.^20^ A recent review on stigma reduction interventions in LMICs identified only nine studies that varied in their educational content, and only three explicitly included stigma sessions.^21^

The deinstitutionalisation of mental health services requires community-level anti-stigma interventions to accompany this process to aid the reintegration of service users within their communities, inclusive of service users themselves, caregivers and their families. Therefore, our study qualitatively evaluated the feasibility and acceptability of a community-delivered mental health intervention for family caregivers from low-income South African communities. For our study evaluation, feasibility is defined as consisting of eight focus areas (i.e. acceptability, demand, implementation, practicality, adaptation, integration, expansion and efficacy testing) in intervention design in the preparation for full-scale implementation.^22^

## Methods

### Study site

The intervention was conducted at an easily accessible central community venue in the Matlosana sub-district of the Dr Kenneth Kaunda District in the North West province of South Africa. The Matlosaana sub-district comprises a population of 417 282 people, serviced by the Tshepong District Hospital, which provides both inpatient and outpatient mental health services in addition to other general and specialist health services.

### Intervention description

An intervention was adapted from a previous psychosocial rehabilitation intervention for service users and their caregivers designed for the South African context^23^ to assist caregivers in coping with the mental health stigma associated with caring for mental health service users. The intervention covered five critical topics for caregivers that addressed common misconceptions about mental illness: general mental health education, mental health stigma, communication and behaviour management, caregiver well-being, and coping strategies. The selection of the topics was based on a recent review of mental health anti-stigma interventions in LMICs,^21^ contextualised and informed by a formative study conducted within our study population.^15^ Development of the training manual and considerations for training facilitators were guided by adult learning principles.^24^

The first topic on general mental health education provided an overview of mental health. The session defined and described mental illness, causes, signs and symptoms, recovery, and medications. The aim of this session was to provide a solid grounding of the basic principles about mental illness, and the session ended with caregivers sharing their experiences with aspects of mental illness presented to promote open and non-discriminatory sharing throughout the intervention.

The second topic, mental health stigma, provided a ‘deep-dive’ into the constructs of mental health stigma and discrimination. The session educated the participants about the myths and misconceptions about mental illness and how these perpetuate fear and stigma. The session concluded by focusing on empowering the participants by teaching them better strategies to cope with mental health stigma.

The third topic, communication and behaviour management, presented the principles of caregiving, effective communication and behaviour management for a mental health service user. Caregivers were taught how verbal and non-verbal communication could show stigmatising attitudes towards mental health service users, leading to poor treatment outcomes and relationships at home. The session encouraged support and promoted mental well-being by learning better ways to communicate and use verbal and non-verbal communication skills. The aim was to ensure caregivers understood that service users’ actions, however inappropriate or uncomfortable, were not intentional, but rather resulted from the disease. Emphasis was placed on better approaches that encouraged empathy and understanding to create a conducive home environment for the service user and the caregiver and/or family.

The fourth topic on coping strategies aimed to give a guide on dealing with common behaviours (e.g. aggression, paranoia, hallucinations, inappropriate behaviour, etc.) of people with psychotic disorders. Caregivers were taught the importance of attentiveness to the signs and symptoms as service users had varied symptoms and behaviours. Lastly, the session taught problem-solving techniques and home environment assessment to ensure safety for persons living with a mental illness.

The fifth and last topic on caregiver well-being acknowledged the burden of taking care of a mental health service user. It aimed to assist caregivers in coping with the needs of service users while preventing caregiver burnout and ensuring that they can cope with the burden of care. Key issues presented included signs of strain and/or stress, identifying and dealing with grief, anxiety and depression, and developing and maintaining mental well-being. This session concluded with sharing information on publicly available mental health support resources and networks.

After the conclusion of the fifth session, time was dedicated to workshop reflections. Conducted as a group discussion, the session aimed to share views and opinions about the intervention and how the information shared may apply to their personal lives. Discussions further extended to the issues that caregivers felt should be raised before concluding the workshop.

A facilitator’s manual was developed to guide the facilitators in delivering the intervention for each topic held during the workshop with the caregivers. The leading facilitator was a clinical psychologist (first author) who developed the intervention and trained a co-facilitator (a registered psychological counsellor) to facilitate the sessions. Face-to-face sessions with caregivers were delivered over three consecutive days.

### Evaluation approach

Framework analysis is commonly used in health sciences and policy research,^25^ and was the qualitative approach adopted in our study. Semi-structured qualitative interviews were used to gain caregiver perspectives regarding their experiences of the mental health anti-stigma intervention and its impact on their coping capacities.

### Sample and sampling strategy

Purposive voluntary sampling was used to select study participants of black African descent from low-income communities. The participants were identified from the health records of the district hospital servicing the Matlosana sub-district where the study was located and invited them to participate. On contacting caregivers, they were invited to participate in our study, which involved a three-day mental health anti-stigma workshop and follow-up interviews two months post-intervention exposure. The workshop for caregivers was conducted in February 2021 at a centralised community venue. A total of 13 caregivers agreed to participate in our study; all 13 caregivers attended all workshop sessions and were interviewed two months post-intervention.

### Data collection methods

Separate interview guides were developed for both caregivers and service users. Caregiver follow-up interviews were structured to discuss issues from mental health literacy, stigma, coping strategies, caregiver well-being and intervention perceptions. Service user follow-up interviews included discussions on mental health stigma, family relationships and coping mechanisms post-intervention. The interviews were conducted by a qualified mental health professional who had experience working with mental health service users and their families. The interviewer took field notes to facilitate the contextualisation of responses during data analysis.

The caregivers and service users were contacted two months after the intervention to elicit their views on aspects of the intervention. In-depth interviews ranging between 45 min and 60 min were held with each caregiver and service user separately. Interviews with both caregivers and service users were conducted in the local language (Setswana) and were audio-recorded.

### Data analysis

Data analysis followed the seven-step framework analysis,^26^ starting with the verbatim translation of audio transcripts from Setswana into English. The researcher familiarised herself with the transcribed interviews and field notes. Caregiver and service user interviews were analysed separately. The researcher developed initial coding frameworks for both datasets based on the interview questions and applied them to the first few interviews while also searching for additional themes emerging to revise the analytical frameworks before application to all transcripts. NVivo 12 was used to apply the analytical framework to all transcripts of both datasets. Data triangulation was done between the caregiver and service user datasets to enrich, explain and confirm study findings regarding the mental health intervention while reducing bias from one participant group’s perspectives. The presentation of study results is aligned to the analytical framework applied to the data. The quotes from the participants were presented with no manipulation by the researchers.

### Trustworthiness

The aspects of methodological rigour (i.e. reliability, credibility, and confirmability) for qualitative studies were evaluated during intervention design. For reliability of study findings, intervention facilitators and interviewers were trained to understand the intervention goals fully. The intervention facilitators were trained on communication skills and encouraging open and non-discriminatory discussions throughout the intervention delivery. The interviewers were trained on the data collection tools, probing techniques and questions standardised to ensure that the responses align with the research objectives. Follow-up interviews were conducted at caregivers’ homes in private, and if home visits were not possible, participants had the option to choose telephone interviews. Both intervention facilitators and interviewers had no prior relationships with our study participants.

For credibility, the interviewers were trained to regularly repeat participants’ feedback, thus ensuring that discussion points were well-understood and not taken out of context. Non-verbal cues were also verbally translated and reiterated to ensure that any misunderstandings were clarified during interviews. The interviewers kept field journals to capture the keynotes that would contextualise participant responses during data analysis and interpretation.

Confirmability of study findings was done through peer checking. Transcribed interviews and field notes were shared with another experienced researcher to analyse, and interpretation differences were discussed to harmonise results.

### Ethical considerations

The study was approved by the University of KwaZulu-Natal’s Biomedical Research Committee under the reference number: BFC 133/19. To access the health facility for our study, the gatekeeper’s approval was obtained from the Department of Health North West province and Witrand Hospital Management. All caregivers and service users provided consent to participate in our study. The research team conducted a standard mental health status check for mental health users to assess stability and coherence before conducting the interviews. The caregivers were reminded that they were free to withdraw at any point during the intervention of follow-up interviews. Personal identifiers in the data were only accessible to the research team that had access to the locked office where data was stored. Any personal identifiers were removed from the analysed data, and names presented in participant quotes have been changed to respect their privacy.

## Results

### Sample demographics

Of the caregivers, the majority were women (*n* = 6), and for most households, average household incomes were below the country’s minimum wage of R3500.00 per month (*n* = 7). The distance to the health facility was reported by most (*n* = 7) as relatively far and very far. The sample characteristics were in line with the research objectives of focusing on low-income communities.

### The overall impression on the intervention

Generally, the intervention was well received and positively regarded by all caregivers (*n* = 10). Positive reviews were centred on the various topics covered during the intervention that most participants felt that had been most impactful to them:

‘It helped me change my behaviour towards my son; I taught my family how he should be treated, and I also spoke to my son, and I see changes in him as well.’ (Caregiver [CG], female, 35 years old)

‘I enjoyed being there with the others because we are in pain over the same thing. Talking about our loved ones was healing, and we also got different ideas or thoughts from the others on how we should take care of them.’ (CG04, female, 50 years old)

‘It helped me change my marriage; learning how to handle people who are mentally ill because they do differ. I was only focused that they are bewitched or overly educated; I didn’t even know that it might run in the family like cancer and other sicknesses.’ (CG08, female, 48 years old)

### Mental health stigma

The caregivers (*n* = 9) felt that their general knowledge and understanding of mental health increased, thus reducing stigma perceptions about service users, mental health, and the mental health facility:

‘I have changed a lot of my negative thoughts that I had towards him.’ (CG02, female, 36 years old)

‘I learned that it’s [*mental illness*] different for everyone, and we end up referring to them as “crazy”, which is not the case.Since the workshop and his return from Witrand, I just take him as a normal person.’ (CG03, female, 51 years old)

‘A lot that changed in me because I would be so angry when talking to him feeling like what he is doing is intentional.’ (CG05, male, 62 years old)

**TABLE 1:** Demographics summary.

Demographics	Caregivers	
	*n*	%
**Gender**		
Female	7	70
Male	3	30
**Ethnicity**		
Black people	10	100
**Age (years)**		
25–30	2	20
31–40	1	10
41–50	4	40
51–60	1	1
61–65	1	1
**Years as caregiver**		
0–2	3	30
3–4	4	40
5–10	3	30
**Average household income**		
< US$240.00	7	70
> US$240.00	3	30
**Distance to a health facility**		
Not far at all	4	40
Relatively far	2	30
Very far	4	40
**Difficulty accessing mental health services**		
Not difficult at all	2	20
Not very difficult	5	30
Very difficult	3	30

Post-intervention, there were increased family cohesion sentiments shared by the caregivers (*n* = 7). It may suggest that the intervention information had been disseminated to other family members in the short term:

‘My family has been very positive; his younger siblings used to say he was crazy. He would get aggressive and want to beat them.’ (CG08, female, 48 years old)

‘Recently, we attended a traditional ceremony at my family’s home … he was happy and dancing.’ (CG04, female, 50 years old)

Community stigma, however, remains a crucial concern for caregivers (*n* = 7), and this suggests a high degree of stigmatisation of people with mental illness in low-income African communities. As an adaptation, it appears that caregivers resorted to ignoring the community’s stigmatising sentiments and actions. While caregivers report that they ignore some stigma experiences, it is clear that they are still affected by these stigmatising experiences and are learning to better cope with them:

‘They [*neighbours*] keep their distance and want nothing to do with him … I only see shame and pity in their eyes, they gossip, and I choose to ignore.’ (CG10, male, 26 years old)

‘The ill-treatment has been there, but we (family) offer her the support that she needs … I ignore them because they don’t know our family situation; they just listen to the rumours.’ (CG01, male, 28 years old)

#### Actions against future perceived mental illness stigma

A majority of the participants (*n* = 6) expressed a desire to challenge the stigmatising views held by people who stigmatise people living with mental illness based on their newfound education.

‘I would try to talk to them and educate them … communicating with them might help so that they can be in my position – understanding how someone with mental illness needs to be treated.’ (CG06, female, 50 years old)

‘I’d reprimand them and ask what if it was their own families, also how is the person meant to come back [*for services*] based on how they are treating them.’ (CG08, female, 48 years old)

Only a few caregivers (*n* = 3) highlighted that they would probably continue to ignore any stigma they experience or witness, which had always been their chosen action as caregivers.

‘I will keep quiet and walk away … I [*currently*] don’t answer them either.’ (CG07, female, 50 years old)

### Influence on caregiver-service user relationships

The caregivers admitted that prior to the intervention, their relationships with service users were strained (*n* = 7). A myriad of factors related to poor relationships were raised:

‘I used to argue with her a lot, and she would want to fight with me even though I tell her that I won’t fight her.’ (CG01, male, 28 years old)

‘I was very short-tempered with him and blamed him that he did this to himself. I would only listen to what he had to say for a short while.’ (CG02, female, 36 years old)

‘I always had a lot to say, and I would shout though I would tell my husband not to raise his voice at him. Once you raise your voice, he gets angry very fast.’ (CG03, female, 51 years old)

Post-intervention, a change in relationships were reported by the caregivers (*n* = 7). The critical issue highlighted was an increased knowledge and insight of mental illness that facilitated their ability to understand better and accept their ward. Furthermore, improvements were highlighted in communication skills and knowing how to manage their service users’ behaviour at home:

‘I have opened up my heart to him; we can sit down and talk, unlike previously. We have both come to terms with everything. He even tells me that he loves me, which he has never done before. At home, he seems more respectful to his grandparents and other children.’ (CG02, female, 36 years old)

‘Our communication has changed, it’s the one thing I failed at. I am not filled with anger when talking to him. I felt overwhelmed and that he was a burden because I had to take care of him and still deal with my life stresses.’ (CG06, female, 50 years old)

‘I now prefer talking to him instead of shouting at him. I used to shout a lot when talking to him, but now my level of understanding is better, though I still shout here and there.’ (CG10, male, 26 years old)

The interviews with service users validated the changes reported by the caregivers. The service users’ perceptions (*n* = 8) strongly suggest that the relationships with their caregivers had undergone positive changes in how they interacted and related to them:

‘She isn’t impatient and now has time for me, seems to understand me a bit better, giving me the benefit of the doubt, and does not want to make me uncomfortable and always tries to encourage me … I was very happy when she showed other people at home how to treat me, and they started changing too.’ (Service user [SU] 01, male, 28 years old)

‘We used to have a lot of conflicts, and he would tell me that I am crazy. It used to hurt me when he said that … We are closer now; he encourages me and tells me that just because I have a mental illness, it does not mean that I am crazy or that I should be pushed aside.’ (SU06, male, 24 years old)

‘I was happy and think she’s more understanding. I think that the workshop has given her more motivation and encouragement.’ (SU08, male, 18 years old)

### Influence on caregiving and burden of care

The caregivers reported a high burden of care before the intervention (*n* = 9). Most of their focus was on ensuring that the service user was well taken care of, especially under limited resources:

‘We don’t care for ourselves; our entire focus is on them being ok and alive.’ (CG06, female, 50 years old)

However, the caregivers reported more collaboration and shared responsibilities between themselves and their service users (*n* = 6). The responses strongly suggest a positive influence of the intervention on reducing the perceived burden of care, allowing the caregivers to have more time for their lives:

‘After the workshop, I gave him a little bit of freedom to see if he would stay on track. I noticed that he stays in his lane, and I don’t need to follow him up constantly.’ (CG03, female, 51 years old)

‘I have a lot of work in the house, so I told her that she could help me with the dishes. I put a chair out for her outside, and she does this while sitting. I don’t want her anywhere; she might hurt herself.’ (CG05, male, 62 years old)

‘I no longer do his laundry and ironing, however when I find that he hasn’t made his bed, I do it for him. He now cleans the house and does the dishes, saying that his siblings just leave the dishes in the sink.’ (CG07, female, 50 years old)

‘I had asked him to clean the yard and his room, which he did at the time but not anymore (service user relapsed).’ (CG10, male, 26 years old)

### Caregiver well-being and coping strategies

The caregivers were appreciative of the intervention’s session on caregiver well-being and coping mechanisms (*n* = 7). A shared concern among the caregivers was the stress associated with caregiving and limited outlets to share their thoughts and concerns. Lessons from the intervention were appreciated for sharing stress relief tips and coping strategies, for example, breathing exercises, buddy system:

‘I loved the part on taking care of ourselves while taking care of our loved ones … When dealing with your own stress and having to take care of someone who is mentally ill just adds to what you are feeling. Instead of assisting with their problems, I add to them because I’d respond with anger.’ (CG06, female, 50 years old)

‘I enjoyed the session on how to take care of yourself to take care of your loved one. You need to keep yourself mentally healthy because if you are both not well, there will be a lot of conflicts.’ (CG08, female, 48 years old)

‘The exercise we were taught when feeling tension, that is beneficial. I always have a lot on my mind, and it has helped me offload some of the thoughts on my mind. Overthinking can be a bad thing, so it helped me a lot.’ (CG10, male, 26 years old)

Throughout the discussions, the issue of financial constraints related to caregiving was raised by the caregivers (*n* = 4). While this was not one of the objectives, the research team allowed the participants to share experiences during the intervention and discuss possible strategies to improve their financial status:

‘I used to work and had to stop working because I feared for the other children at home. My husband is the only one working …and we are struggling. His younger brother is now in matric, and we are not sure what will happen with his education next year.’ (CG03, female, 51 years old)

‘If she can get a wheelchair or crutches, that will help when we go to the hospital. She has a challenge with being mobile; we spend an hour just to get to the nearby shop.’ (CG05, male, 62 years old)

### Perceived intervention facilitators and barriers

The group sessions appealed to the caregivers (*n* = 10), and the feedback suggests this was a vital driver of the intervention’s effectiveness:

‘I found comfort and realised that I had to be more accepting because I wasn’t the only one going through this. They made me realise that I still have a long life to live with Sarah.’ (CG04, female, 50 years old)

‘It was very nice; it was the first time that I experienced such (a workshop). It was good to hear from others also how to take care of someone who is mentally ill … During lunch, we had a chance to talk and share what others do that works for them.’ (CG05, male, 62 years old)

‘It was easier to open up because people there understood what I was going through. It’s unlike talking to someone who doesn’t seem to understand, so I was happy to be around them.’ (CG06, female, 50 years old)

Although perceived barriers were few, some caregivers (*n* = 3) felt that including other healthcare professionals (e.g. doctors, pharmacists, social workers, etc.) would be beneficial in enriching some of the discussions (e.g. medications, managing and caring for service users):

‘I would have loved if there was a doctor. I wouldn’t expect a psychologist to know deeper information concerning medications. She deals with people’s emotions, not medication.’ (CG08, female, 48 years old)

### Suggestions for future interventions

#### Extended counselling

The caregivers believed that group sessions facilitate open dialogue and sharing of experiences (*n* = 2). However, it was suggested to consider the addition of individual counselling sessions for those who require it:

‘I think group and one-on-one sessions options are good because someone might have a different idea when questions are being asked.’ (CG01, male, 28 years old)

#### Awareness campaigns to the larger community

While the caregivers reported a better understanding of mental illness post-intervention, they recommended community awareness initiatives to eliminate mental health stigma (*n* = 6). These initiatives would assist the successful reintegration of mental health service users, reducing the need to restrict them:

‘I think if there’s awareness in the communities, this might make things easier for those who have locked up their loved ones because they don’t know what to do with them.’ (CG06, female, 50 years old)

## Discussion

The responses from the caregivers who participated in the feasibility study strongly suggest the acceptability of the intervention. The caregivers reported satisfaction and endorsed the intervention as helpful in caring for the service user and themselves. Self-reported positive changes in mental health stigmatising attitudes among the caregivers and subsequent improvements in relationships with the service users were attributed to the intervention. The caregivers were appreciative of how the intervention helped them understand mental illness, and in turn, caregivers shared their newfound knowledge with their immediate family members. Similar results of acceptability have been reported from other mental health interventions for caregivers and their families.^27,28,29^

The intervention was delivered from a relatively accessible central venue in the community. Participation and completion of the intervention remained throughout, suggesting the acceptability and utility of the community-based support group approach. The feasibility and acceptability of group-based mental health interventions and their effectiveness to improve clinical outcomes have been successfully demonstrated in the case of other public health issues in the region, such as HIV or AIDS in Tanzania.^30^ For broader application at scale, training existing community-based workers such as auxiliary social workers under the supervision of social workers to run the support group sessions for service users and caregivers within communities should be considered and evaluated accordingly. Task-sharing of responsibilities for mental health services has been proven feasible and effective in LMIC countries such as Zimbabwe where there are chronic shortages of health workers.^31,32,33^

For future applications, several considerations need to be made. The caregivers suggested the inclusion of support options (individual and group sessions) and medical personnel to enrich the intervention. These suggestions are probably unrealistic in the context of sparse medical and mental health specialists in South Africa. The inclusion of a session on common medications prescribed for mental health service users with severe mental illness and about their side effects should be considered for similar programmes in the future. Referral pathways to existing mental health professionals should also be included for those requiring additional professional support.

The advent of the coronavirus disease 2019 (COVID-19) pandemic disrupted health services and had an overwhelming impact on mental health distress (Moitra et al. ; Yao et al.).^34,35^ Not only has it foregrounded the need for strengthened mental health services globally, but it has also highlighted the need for digital platforms to provide workshops and training to promote social distancing. Previous studies in high-income countries have assessed the feasibility and accessibility of digital approaches to mental health interventions through telehealth and concluded that the approach could be widely applied.^36^ There is increasing evidence of the efficacy and potential of telehealth interventions for mental illness and marginalised groups in developing countries.^37,38^

The caregivers recommended that future interventions consider community awareness initiatives to improve mental health literacy and reduce mental health stigma within communities to improve the social landscape for people living with a mental illness. Understandably, community stigma remains a vital issue of concern for both caregivers and service users. Failure to address the social stigma around mental illness threatens the social reintegration efforts of persons living with mental illness, especially in African settings where stigma remains high.^7,10,13^ This insight of caregivers on the need for community awareness interventions to reduce mental health stigma at a population level needs emphasis as community care demands increased social acceptance of people living with a mental illness.

Our study noted substantial financial distress among the families of mental health service users. Our study participants were from low-income communities, and their demographics showed that the majority have monthly household incomes that fall below the South African minimum wage of approximately US$240.00. Mental health stigma in low-income South African communities has been high.^4,39^ In an already financially constrained setting where misconceptions on mental illness are prevalent, the need for stigma reduction strategies needs to be emphasised. While interventions such as the current one may contribute to reducing stigma, the financial burden of care also requires attention, and strategies need to be developed to assist caregivers in improving and strengthening their financial independence.

## Limitations

The small sample size and the qualitative nature of the study limit the generalisability of the findings to the broader South African population. One referral health facility was used to conduct the study, limiting the applicability of results to the specific population [and ethnic] group where the study sample was drawn.

Regarding the study sample used in the analysis, two caregivers were excluded from the data analysis because their service users had recently deceased. One caregiver could not be reached for follow-up, resulting in ten caregiver follow-up interviews. In addition to the caregiver interviews, nine of the service users cared for by the caregivers were interviewed. One could not be interviewed because they had relapsed and were hospitalised.

### Conclusion

Our study demonstrates that a group-based intervention involving caregivers and mental health service users holds potential for helping to reduce stigma, strengthening relationships between caregivers and mental health service users, and promoting coping and alleviating some aspects of the burden of care. With the South African government’s drive for deinstitutionalisation of mental health services, the need to integrate such community group-based initiatives that address the mental health stigma of caregivers within community mental health services is essential to increase the uptake of services and facilitate social reintegration of service users. However, the need for population-based anti-stigma interventions is also required to facilitate social reintegration and acceptance of mental health service users within communities.
